# Two-stage hepatectomy for multiple giant alveolar echinococcosis

**DOI:** 10.1097/MD.0000000000007819

**Published:** 2017-08-18

**Authors:** Hao-De Shen, Ke-Fei Chen, Bo Li, Han-Zhi Zhang, Kang-Ming Yang, Yin Chen, Jia-Xin Li, Zhe-Yu Chen, Ta Meng, Zhi Ma, Hong-Zhi Li

**Affiliations:** a Department of Liver Surgery and Liver Transplantation Center, West China Hospital of Sichuan University, Chengdu; b Hydatid Disease Institute of Ganzi Prefecture, Ganzi Tibetan Autonomous Prefecture, Sichuan Province, China.

**Keywords:** alveolar echinococcosis, insufficient future remnant liver volume, liver regeneration, two-stage hepatectomy

## Abstract

Alveolar echinococcosis is a chronically progressive and potentially fatal disease. Patients with multiple giant alveolar echinococcosis have a poor prognosis when radical resection cannot be achieved, but curative resection can be limited by low future remnant liver volumes. In these cases, 2-stage liver resection may be a better choice: after a first-stage hepatectomy with partial resection, liver regeneration is allowed in the residual liver before proceeding to the second-stage hepatectomy. In this study, we therefore retrospectively reviewed and evaluated the safety and feasibility of two-stage hepatectomy in patients with multiple giant alveolar echinococcosis.

We reviewed the data for all patients who underwent 2-stage hepatectomy for multiple giant alveolar echinococcosis between August 2013 and December 2015 at either the West China Hospital of Sichuan University or the Hospital of Ganzi Tibetan Autonomous Prefecture.

We identified 7 patients in whom 2-stage hepatectomy was completed. During the first-stage hepatectomy, 4 patients underwent right-sided hepatectomy and the other 3 underwent left-sided hepatectomy. The second-stage hepatectomies were successfully performed 3 months after the first-stage procedures. All patients had follow-up durations of >1 year; there were no cases of operation-related mortality, and no patients experienced disease recurrence.

Two-stage hepatectomy is safe and feasible for patients with multiple giant alveolar echinococcosis.

## 1 Introduction

Echinococcosis or hydatid disease is a zoonosis caused by the larval stages of tapeworms that can severely impair human health. The liver is the main site of disease, both for alveolar echinococcosis (AE) and cystic echinococcosis,^[1]^ with the former being more severe and more difficult to treat than the latter. AE is also difficult to detect at an early stage, and lesions are typically large, multiple, or even infiltrating adjacent structures at the time of initial diagnosis. Indeed, AE displays a tumor-like infiltrative behavior, and about 90% of untreated patients will die within 10 years of detection.^[2]^ Surgery is the first-choice option whenever feasible,^[3,4]^ but radical resection is only possible in 35% of patients.^[5]^ Liver transplant, which may be the only curative treatment option, has therefore been employed for unresectable liver disease.^[6,7]^ Unfortunately, the shortage of donor livers, high cost of treatment, and other important issues limit the use of transplantation. Preoperative volume measurement is a good way to estimate the future remnant liver volume (FRLV) accurately, with evidence showing a high risk of postoperative liver failure after 1-stage resection when patients had a low FRLV (i.e., <30%).^[8]^ Two-stage liver resection may be a good choice when patients have multiple huge lesions because of advanced AE, with the possibility that the first-stage procedure can improve future operability and reduce the associated operative risks at the second operation. In this article, we present our experience using 2-stage hepatectomy for the treatment of multiple huge AE disease.

## 2 Methods

### 2.1 Study design

We retrospectively collected data related to patients treated for multiple hepatic AE by 2-stage liver resection at either the West China Hospital of Sichuan University or the Hospital of Ganzi Tibetan Autonomous Prefecture. The study setting was in Sichuan Province, and it was conducted between August 2013 and December 2015.

### 2.2 Patient selection

Patients were selected based on the following inclusion criteria: diagnosis of AE; age <60 years; patients can reach radical resection; preoperative volume measurement with a predicted FRLV <30%; Child–Pugh class A; and no history of therapy before the first-stage operation. Patients were not offered 2-stage surgery if they met the following criteria: diagnosis of liver cirrhosis; main portal vein or inferior vena cava invasion; lesions were cystic; any immunodeficiency or autoimmune disease (e.g., rheumatic arthritis, Buerger disease, multiple sclerosis, or type 1 diabetes mellitus); any organ failure; or mental illness. All selected patients consented to treatment.

### 2.3 Surgery

Careful evaluation was necessary before every operation, with the aim being to remove partial lesions at the first operation. The interval from the first to the second operation was approximately 3 months, and residual lesions were assessed for removal at the second operation if sufficient regeneration of residual liver volume had occurred. Hepatic reserve function was evaluated by FRLV, using computed tomography (CT). For the second operation to proceed, the FRLV needed to be >35% and the indocyanine green retention rate after 15 minutes needed to be <10%. In all cases, sufficient evaluation was done to assess for aberrant vascular anatomy, vessel invasion, and features of vasculature conjunction.

**Figure 1:**
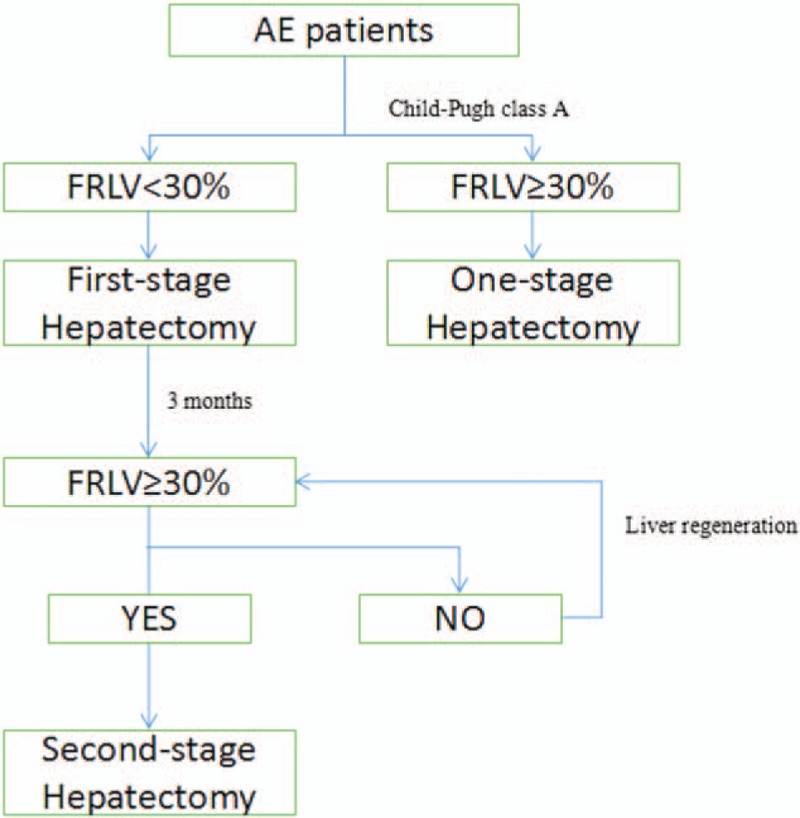
A flow diagram summarizing the two-stage hepatectomy procedure.

A right subcostal incision was used in the first operation. After adequately freeing the liver on that side, the intraperitoneal cavity was explored by bimanual palpation and intraoperative ultrasound to establish the extent of disease. To avoid extensive adhesions at the second-stage surgery, conservative mobilization of the liver was done, ensuring minimal division of the falciform ligament and diaphragmatic attachments. A sling was placed around the hepatoduodenal ligament such that it would not tighten until blood loss exceeded 200 mL. For the liver resection, a clamp-crushing technique and UltraCision harmonic scalpel were used. Vessels with diameters <3 mm were closed by harmonic scalpel, proximal intrahepatic ducts measuring 0.3 to 1 cm were ligated, and distal ducts were clipped with titanium clipping. Vessels >1 cm in diameter were sutured with 5–0 vascular slide wires. The second operation was performed 3 months after the first operation and was done through the previous incision. Abdominal adhesions after hepatectomy for AE are usually very dense, so the first step of the second operation was to separate the adhesions. Beyond that, the procedures were the same as for the first operation. Figure 1 illustrates the above process in a simplified flow diagram.

### 2.4 Follow-up and long-term management

Long-term albendazole was mandatory after both surgical treatments (at least 6–12 months).^[9]^ For this, albendazole was given orally at a dosage of 15 mg/kg/day in 2 divided doses. Routine blood tests were monitored during medical therapy. During follow-up, CT and liver function tests were repeated every month after the first-stage surgery. Thereafter, patients were followed up at 1 month, 6 months, 12 months, and every year after the secondary operation. This included CT or magnetic resonance imaging (MRI), liver function tests, routine blood tests, and hydatid antibodies.

### 2.5 Statistical analysis

Statistical analysis was performed with IBM SPSS Version 21.0 (IBM Corp, Armonk, NY). Normally distributed continuous variables are presented as means ± standard deviations, and variables without a normal distribution are presented as medians. Categorical variables are presented as frequencies.

## 3 Results

### 3.1 Patient data

We collected data for 7 patients with multiple hepatic AE who were treated by 2-stage liver resection during the study period and who met our inclusion criteria. Table 1 shows the clinical and demographic characteristics of the 4 males and 3 females we included (mean age 35 years, range 21–52 years).

**Table 1:**
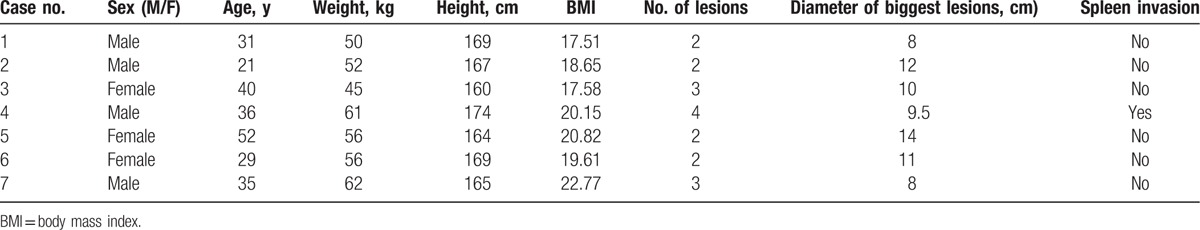
Clinical features of the patients.

### 3.2 Clinical presentation

**Figure 2:**
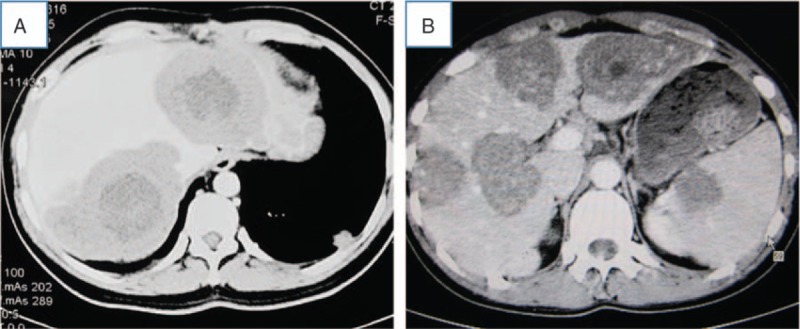
Representative abdominal computed tomography images of alveolar echinococcosis. (A) Abdominal computed tomography showing a patient with two huge alveolar echinococcosis lesions in the right and left lobes. (B) Abdominal computed tomography scan showing a patient with 4 big liver lesions and a metastatic lesion in spleen.

The most common symptom at diagnosis was nonspecific upper abdominal pain. Preoperative diagnosis was considered multiple hepatic AE in all cases, and according to the PNM (P = parasitic mass in the liver, N = involvement of neighboring organs, and M = metastasis) hepatic AE staging classification, the cases had stage IV disease.^[10,11]^ CT or MRI showed changes typical of multiple hepatic AE, and all patients had positive blood tests for hydatid antibodies. Both the left and right lobes of the patients’ livers were involved. Four patients had 2 huge lesions located in both the left and right lobe of their livers (Fig. 2A), 2 cases had 3 huge lesions in their livers, and 1 patient had 4 alveolar lesions with splenic invasion (Fig. 2B).

### 3.3 The first operation

At the first operation, 4 patients underwent treatment to remove right-sided liver lesions and 3 underwent surgery to remove left-sided lesions. After surgery, the first 3 cases underwent spiral CT every month to calculate their post-surgery residual hepatic volumes; all 3 cases reached an FRLV of at least 35% by 3 months after surgery. However, liver volume measurement was repeated in the other 4 patients at 3 months after the first surgery. The second resections were only performed after satisfactory evaluation.

**Figure 3:**
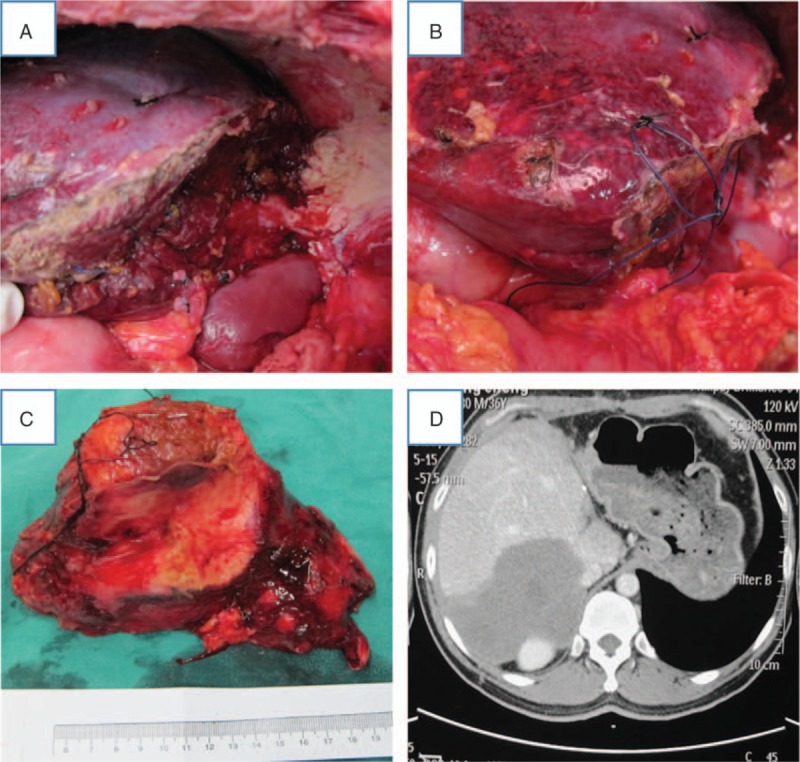
A representative case during and after first-stage hepatectomy. (A) The cut surface of the liver during hepatectomy. (B) The 7–0 silk suture placed around the hepatoduodenal ligament after the first resection. (C) The resection specimen. (D) Computed tomography image before the second-stage hepatectomy.

An example of the first-stage operation can be given for a patient with 2 huge AE lesions who underwent left hepatectomy (Fig. 3A). In this patient, a 7–0 silk suture was kept around the hepatoduodenal ligament after the first resection, which is necessary to enable the hepatoduodenal ligament to be found easily at the second operation when the hilum is involved (Fig. 3B). As shown, the lesion in the left lobe of liver was removed at the first operation (Fig. 3C), and postoperative CT showed that the liver had good compensatory regeneration (Fig. 3D).

The median time of the first operation was 181 minutes (135–297 min), and 4 of the 7 cases required inflow hepatic vascular occlusion for a median time of 21 minutes (14–35 minutes). The median blood loss was 290 mL (150–400 mL) and no blood transfusions were needed. Postoperatively, liver function (including total bilirubin, aspartate transaminase, and alanine transaminase) recovered to normal levels within 7 days in all 7 cases. The median length of hospitalization was 10.8 days. There were no operation-related deaths and no serious complications (Table 2).

**Table 2:**
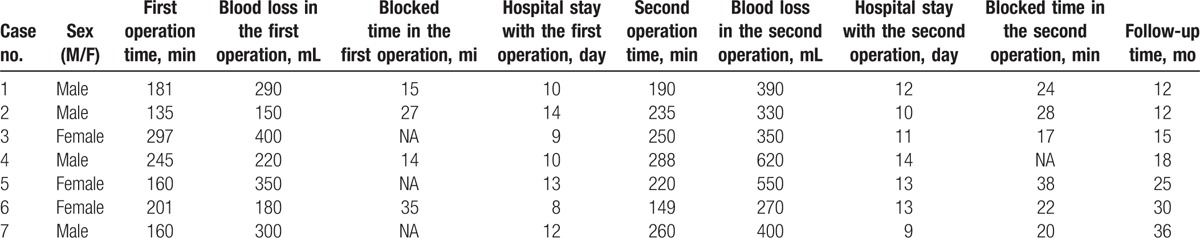
Intraoperation and post operation features of the patients.

### 3.4 The second operation

**Figure 4:**
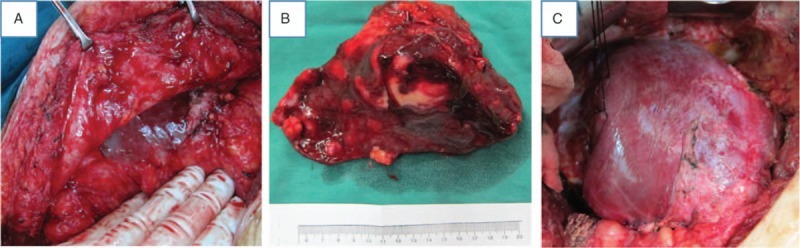
A representative case during the second-stage hepatectomy. (A) Severe adhesions can be seen around the liver. (B) The resection specimen. (C) The cut surface of the liver after the second-stage hepatectomy with evidence of compensatory regeneration in the liver remnant.

**Figure 5:**
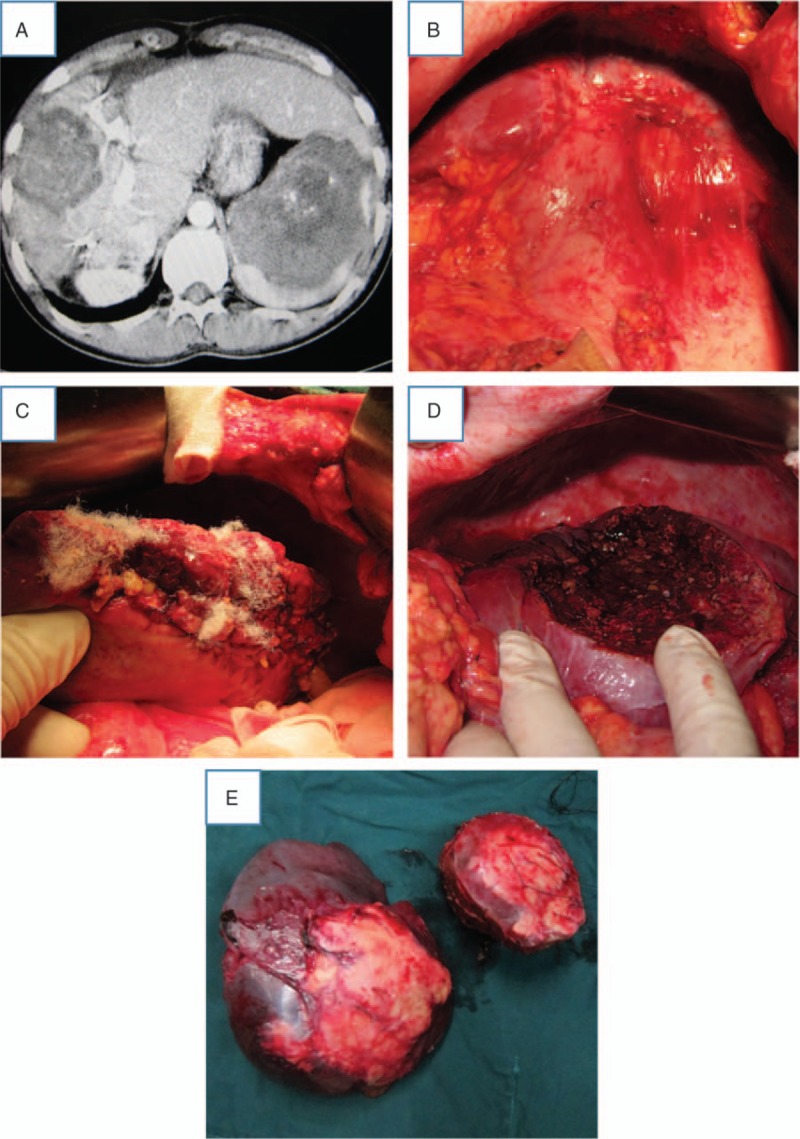
Second-stage hepatectomy in a patient with four huge alveolar echinococcosis lesions and a metastatic lesion in spleen. (A) Computed tomography before the second hepatectomy. (B) There is evidence of extensive abdominal adhesions and liver regeneration. (C) Image of the left cut surface of the liver after second hepatectomy. (D) Image of the right cut surface of the liver after second hepatectomy. (E) The 2 specimens from the second-stage procedure.

After separating the adhesions that resulted from the first operation, the second operation proceeded in the same way as the first (Fig. 4). An example of a second-stage operation can be given for a patient with 4 huge AE lesions and a metastatic lesion in the spleen. In this case, we resected the lesions in the middle of the liver in the first operation, which allowed good regeneration at this site and laid the foundation for the second operation. The remaining hepatic lesion could then be successfully resected in the second operation (Fig. 5).

The median time for second operation was 235 minutes (149–288 minutes), and 6 of the 7 cases suffered inflow hepatic vascular occlusion for a median time of 24 minutes (17–38 minutes). The median blood loss was 390 mL (270–620 mL) and no blood transfusions were needed. Postoperatively, liver function (including total bilirubin, aspartate transaminase, and alanine transaminase) recovered to normal within 7 days and the median length of hospitalization was 11.7 days. However, 1 patient developed bile leakage, but this was managed conservatively. All AE lesions were fully removed and the diagnoses were confirmed by pathological examination. Finally, all patients received follow-up of >1 year, during which time there was no operation-related mortality or disease recurrence (Table 2).

## 4 Discussion

AE is endemic in many regions of the world, but especially in parts of the Northern hemisphere, including North America, China, and some European countries.^[12–14]^ The annual incidence in endemic areas of Europe increased from a mean of 0.10 per 100,000 in 1993 to 2000 to a mean of 0.26 per 100,000 in 2001 to 2005,^[15]^ indicating that AE is posing an increasing disease burden. Moreover, most patients with hepatic AE have advanced hepatic disease when first diagnosed,^[16]^ and drug therapy alone is usually ineffective when patients present with giant hepatic AE. In these cases, radical surgical resection is the most effective treatment.^[17]^

Hepatectomy can be considered safe if the texture of the remnant liver is normal and the residual liver volume is >35%. However, if the residual liver volume is 3 < 5% (or even 3 < 0%), then the risk of surgery is high. In general, when treating multiple giant hepatic AE, even if the remaining liver texture shows no invasion by hydatid, hepatectomy should not be considered if the FRLV is 3 < 5% or 3 < 0%.^[18]^ For those patients with “unresectable” but nonmetastatic AE lesions, liver transplantation is usually considered, but the high cost, shortage of liver donors, risk to life (including acute liver failure owing to small-for-size syndrome), and need for long-term immunosuppressive therapy limit reliance on it for routine therapy. Ex-vivo liver resection and autotransplantation have also been done in some centers^[19]^; however, this operation is unsuitable when the patient has an FRLV 3 < 5% and graft volumes are insufficient. This is important because the degree of injury caused by ex-vivo liver resection and autotransplantation are high, as are the postoperative complication rates.

To overcome the problems of existing medical and surgical therapy, 2-stage hepatectomy has been introduced and used successfully in some cases. The major indications for 2-stage hepatectomy are an FRLV 3 < 5% and no lesion invasion into the hepatic hilum. In this study, one of our patients developed splenic metastasis, but radical treatment was still possible by 2-stage hepatectomy. Therefore, extrahepatic metastasis should not be considered a contraindication to hepatectomy for AE if the lesion is completely resectable.

Two-stage hepatectomy appears to be particularly suited to patients with AE. This is because the liver has a very strong regenerative capacity, which we can make use of through staging operations that make lesions resectable within the FRLV limits. Based on our experience reported in this article, staging surgery allows removal of the lesions that are most problematic that are most likely to invade the first and/or second hepatic portal after the first-stage operation. If lesions can be easily resected at the first operation, and if the most difficult lesion cannot be removed at the second operation, then there is no need for a staging operation to treat AE. It is possible that if the lesions most likely to invade the first and/or second hepatic portal are not removed at the first operation, invasion could occur while waiting for the second operation. If this happens, the prognosis would be poorer and the difficulty of any subsequent operation would increase.

When regeneration of the remnant liver has reached the point where the volume exceeds 35%, we can consider the second-stage operation. This equates to a time interval between the 2 stages of about 3 months because this is usually sufficient to reach a suitable volume of regeneration. A balance must be maintained, however, because AE can still advance slowly during the interval between the 2 operations.

In conclusion, we showed that 2-stage hepatectomy is safe and feasible for patients with multiple giant AE. After follow-up, it was shown that the 2-stage operation was effective for removing lesions in all 7 patients. To date, radical cure has been achieved in all cases without severe complication, death, or recurrence. Therefore, the 2-stage method has the potential to have less risk and greater success rates when used for the treatment of complex giant hepatic AE. Further clinical studies are needed that include larger samples.
